# Sleep Duration as the Main Indicator of Self-Rated Wellness and Health among Healthcare Workers Involved in the COVID-19 Pandemic

**DOI:** 10.3390/ijerph19010136

**Published:** 2021-12-23

**Authors:** Maryam Masoumi, Kamyar Shokraee, Somayeh Mohammadi, Soroush Moradi, Mohammad Bagherzade, Javad Balasi, Abbas Smiley

**Affiliations:** 1 Department of Internal Medicine, Qom University of Medical Science and Health Services, Qom 37169-65384, Iran; m.masoumiy@gmail.com (M.M.); s.mohammadi199620@gmail.com (S.M.); 2 Department of Surgery, Iran University of Medical Sciences, Tehran 14496-14535, Iran; k-shokraee@alumnus.tums.ac.ir; 3 Department of Internal Medicine, Tehran University of Medical Sciences, Tehran 14176-53911, Iran; moradi-s@alumnus.tums.ac.ir; 4 Shahid Beheshti Hospital, Qom University of Medical Sciences, Qom 37169-65384, Iran; m_bagherzadeh3@yahoo.com; 5 School of Medicine, Iran University of Medical Sciences, Tehran 14496-14535, Iran; Javadbalasi8@gmail.com; 6 Westchester Medical Center, New York Medical College, New York, NY 10595, USA

**Keywords:** COVID-19, pandemic, healthcare workers, lifestyle, sleep

## Abstract

Objective: This study was performed during the COVID-19 pandemic to better understand the indicators of self-rated wellness and health among healthcare workers. Methods: Sleep pattern, mood status, nutritional condition, physical activity, habits and the subjective wellness and health index of the healthcare workers of a university affiliated hospital were surveyed. Paired *t*-tests were performed to compare the participants’ quality of life before and after the outbreak of COVID-19. Multivariable linear regression models with a backward elimination stepwise process determined the parameters that significantly correlated with self-reported wellness and health. Results: Of the 200 healthcare workers who participated in this study, 119 (60%) were female and 81 (40%) were male, with a mean (SD) age of 28.8 (5.9) years. We found that the COVID-19 pandemic significantly changed many lifestyle factors compared to the pre-pandemic states. The scores of sleep quality, mood status, pre-planned physical activity and social activity were reduced by 30%, 40%, 50% and 70%, respectively. The average night sleep duration before the pandemic was 7 h and 22 min, whereas during the pandemic it decreased to 6 h and 44 min, a debt of 38 min in sleep duration every night. As found by multivariable regression modelling, self-reported wellness and health before the pandemic period was associated with wake-up time, mood status, physical activity and diet. During the pandemic period, in addition to these variables, night sleep duration (β = 0.049, *p* = 0.049) and nap duration (β = 0.009, *p* = 0.01) were left in the final multivariable model and correlated significantly with the wellness and health index. Conclusion: COVID-19 has detrimentally affected healthcare workers’ well-being and quality of life. Sleep duration was the main factor correlated with subjective wellness and health index during the current COVID-19 pandemic.

## 1. Introduction

Following the emergence of SARS-CoV-2 in December 2019 and the pandemic it caused, healthcare workers’ health and well-being came to the spotlight. On the one hand, the drastic change in workplace risks and the tension and anxiety associated with them [1], and on the other hand, the social pressure (and in some contexts, the social exclusion) caused by the unquestionable role of medical professionals in the current pandemic state, threatens their mental health as individuals and their quality of care as professionals [2]. The fear of being a threat to the well-being of vulnerable family members, stigmatization of the infected, heavy restriction of workplace flexibility, and strict measurements for disease prevention such as protective masks and gowns all contribute to an increase in depression symptoms and a decline in quality of life among those working in the medical setting [3,4]. While many questions about COVID-19 remain unanswered, healthcare workers’ well-being in the face of additional workload and increased workplace-associated risks remains a priority. The outcome of this pandemic depends heavily on the medical system’s efficiency and maintaining the well-being of healthcare workers is essential to their effectiveness [5]. Being on the lookout for possible signs of excessive pressure among staff and a better understanding of the pandemic’s effects on their lifestyle can guide us toward improving their quality of life. Different elements of lifestyle may compromise the wellness and health of healthcare workers. Physical activity [6,7,8,9,10,11,12], nutrition [13,14], smoking [15] and mood [16,17,18,19] all contribute to their well-being and health status by various degrees and different mechanisms. One of the most important elements of lifestyle is sleep. Chronic sleep debt has been shown to negatively affect wellness and health, as well as healthcare worker performance in terms of clinical errors, mood, motivation, creativity and leadership [20,21,22,23]. Herein, we aimed to investigate the lifestyles of healthcare workers during the COVID-19 pandemic and study the parameters that contribute to wellness and health as reported by them.

## 2. Methods

### 2.1. Participants

This cross-sectional study was performed on the healthcare workers of a university-affiliated hospital including interns and nurses. The university ethics committee approved the protocol of study. Participants were asked to provide a written consent form before their enrolment.

### 2.2. Measurements and Data Collection

In addition to demographical information, a questionnaire addressing subjects’ quality and quantity of sleep, mood status and nutritional condition was prepared according to the Mini-Sleep Questionnaire, Gallup Well-Being Index, and Gallup Diet Questionnaire, respectively (Table 1) [24,25,26]. Nineteen questions evaluated sleep, of which nine were related to sleep duration and ten to sleep quality. Mood status was assessed by five questions, and the nutritional condition was addressed by three other questions. Participants were required to answer each question based on the event’s frequency, with 0 meaning that the complaint never happened and 7 meaning that the complaint was present during every day of the week. The sum of the scores of all questions in each part equaled the overall score of each part. A higher overall score indicated a worse quality of life. A similar format of social support questions was constructed [27]. Participants’ physical activity was asked about using a modified question from the Brunel Lifestyle Physical Activity Questionnaire [28], indicating their physical activity level per week. Additionally, to account for the detrimental effects of smoking and alcohol on participants’ health and wellness, they were questioned in subsequent parts on their level of consumption [29]. Finally, the subjects were required to rate their own wellness and health on a scale of 0 to 20, with 20 being as healthy as possible and 0 being as unhealthy as possible [30]. Table 1 summarizes all the questions with due attention to the section they evaluate.

## 3. Statistical Analysis

The frequency distributions of demographic information, wellness and health parameters, lifestyle elements and comorbidities were determined using descriptive analyses. Leven’s test was used to assess the normal distribution of the variances. A *t*-test was used to compare continuous variables between males and females because lifestyle factors and self-image could have been significantly different between males and females [31]. A paired *t*-test was used to compare continuous variables between the pre-pandemic and current pandemic periods. Multivariable association of the self-rated wellness and health index and other assessments was assessed through linear regression analysis with a backward stepwise elimination process. Data analyses were conducted using SPSS (version 26, Chicago, IL, USA). A *p*-value of <0.05 was considered significant.

## 4. Results

A total of 200 healthcare workers aged 22–50 years old were enrolled in the current study. Their mean (SD) age was 28.8 (5.9) years. A total of 119 subjects were female (60%). The lifestyles of participants are summarized in Table 2. Overall, most elements of lifestyle became worse during the COVID-19 pandemic compared to the pre-pandemic period. Sleep quality, mood, physical activity and social activity were 30%, 40%, 50% and 70% worse in the pandemic period compared to the pre-pandemic states, respectively. The mean time to go to bed and wake-up time on weekdays in the pandemic were 35 and 24 min later than those in the pre-pandemic period, respectively. The mean time to go to bed and wake-up time on weekends in the pandemic were 21 and 6 min later than those pre-pandemics, respectively. The self-rated wellness and health index also decreased by 4% during the current pandemic. The only observed improvement was in the smoking rate (Table 2).

Before the pandemic period, subjects went to bed on weekends 40 min later than on weekdays (*p* < 0.0001). In the pandemic period, this difference decreased to 26 min (*p* < 0.0001). In both the pre-pandemic and pandemic periods, subjects woke up 96 min later on weekends compared to the weekdays (*p* < 0.0001). Before the pandemic, the mean sleep duration every night was 7 h and 22 min for the weekdays, which decreased to 6 h and 44 min during the pandemic. The difference signified a 38 min sleep debt every night. The night sleep duration on weekdays before the pandemic was 33 min shorter than the night sleep duration on weekends (*p* < 0.0001). This difference decreased to 2 min during the pandemic. The mean nap duration was more than one hour, whether on weekends or weekdays, in both the pandemic and the pre-pandemic periods.

Females and males were compared in terms of different lifestyle elements. Although the smoking rate was always significantly worse in males than females, the following lifestyle elements were significantly worse in females than males in both the pre-pandemic and pandemic periods: sleep quality, mood, and physical activity (Table 3). There was no significant difference between females and males in most sleep duration variables. The only exception was the nap duration on weekdays in the pandemic period, which was significantly longer in females than in males (Table 3).

A total of 17 subjects had confirmed COVID-19 by swab test, of which 10 were female (59%) and 7 were male (41%). The most common accompanying disease was hypothyroidism (5.5%). Table 4 demonstrates the multivariable linear regression models of the self-rated wellness and health index, compared with the pre-pandemic and pandemic periods. The following variables were left in the pre-pandemic regression model by backward elimination: sex, mood status, physical activity, diet and wake-up time. In the multivariable model built for the pandemic period, mood status, physical activity, diet and wake-up time stayed in the model, but sex was replaced by two other sleep quantity variables, i.e., night sleep duration on weekdays and nap duration on weekdays. The strength of association of pre-planned physical activity and the wellness and health index in the pandemic period was double of that in the pre-pandemic period (Table 4).

## 5. Discussion

By comparing the two regression models of the pre-pandemic and pandemic periods, we were able to detect a pattern of significance of sleep duration for the pandemic period model. In our final regression model for the pre-pandemic period, sex, mood status, physical activity, diet and wake-up time were the principal indicators of the wellness and health index, whereas during the pandemic period, two sleep duration variables showed significant associations as well. In practice, however, the heavy burden of the pandemic on the medical system has led to a significant decrease in the participants’ sleep quality, mood status and physical activity compared with the pre-pandemic states by 30%, 40% and 50%, respectively, as shown in Table 2. The mean sleep quality score before the pandemic was 11.84 out of 70, which became worse during the pandemic (15.24/70). Similarly, the average night sleep duration during the week was 7 h and 22 min before the pandemic, which decreased to 6 h and 44 min during the pandemic. Sleep quality was also evaluated. It became worse during the pandemic compared to the pre-pandemic state. It was also entered in the multivariable linear regression model, which was removed by a backward elimination process. These series of broken routines and vicious circles have led to Coronasomnia. Although insomnia among healthcare professionals was a serious problem before the COVID-19 pandemic, it is now an enormous disruptive lifestyle factor. The 28% decrease in quality of sleep along with 38 min of sleep debt during the pandemic period compared to the pre-pandemic state could be the main indicators of health-related well-being in the pandemic period. This finding has been highlighted by the backward elimination multivariable linear regression model. Thirty-eight minutes of sleep debt every night can dramatically deteriorate the well-being, health and performance of healthcare workers in the long term. This is especially important during the pandemic period, the shortage of workers along with high patient loads leading to the fatigue of healthcare workers. Given the current pandemic and the chronic sleep debt, it may take longer to recover than one may think [20,22]. In line with our findings, a study of 1563 healthcare workers during the COVID-19 outbreak found that more than one-third of healthcare workers suffered from insomnia [2]. Sleep insufficiency among healthcare practitioners has been reported by several studies during the current pandemic [32,33,34]. In another study, 38% of pediatric healthcare workers suffered from sleep disturbances, and 20% showed anxiety and depression symptoms [34]. Multiple studies conducted in different parts of the world have shown similar findings among frontline healthcare professionals who are actively fighting COVID-19 [35,36,37,38,39,40,41,42,43,44]. In the current medical setting, sleep quality and quantity among healthcare professionals are now much more attention-worthy. Poor sleep can decrease professional performance and increase medical errors and burnout, as demonstrated by various studies [45,46,47,48]. It can also disrupt the normal functions of the immune system, resulting in increased susceptibility to COVID-19, which in turn will further increase the pandemic’s burden on the medical system and reduce the quality of care [49,50]. In addition, sleep insufficiency may lead to more severe anxiety and depression [51]. Mood status was another main indicator of self-rated wellness and health status in our study. Long-term effects of sleep insufficiency are not limited to mood status. Obesity, dyslipidemia, hypertension, diabetes, cardiovascular diseases, cancers, cognitive decline and Alzheimer’s disease are some of the most serious long-lasting consequences of a poor sleep habit [52,53,54,55] (Liu et al., 2013; Smiley et al., 2019b, 2019a, 2018).

Our study’s critical shortcoming was the limited applicability of the self-reported wellness and health index and lifestyle factors in assessing the quality of life of the healthcare professionals; thus, we encourage objective methods of recording staff’s wellness and health and their lifestyle in studies to come. The COVID-19 pandemic occurred unexpectedly, and it was not possible to conduct a follow-up study to assess the influence of the pandemic on lifestyle before and after the pandemic. The study was conducted during the COVID-19 pandemic period and required information regarding the current lifestyle during the pandemic period as well as the lifestyle before the pandemic period. The retrospective design was the only way to gather pre-pandemic data about the sample and compare it with the pandemic data; however, we acknowledge the limitations of the retrospective design in terms of it being inferior evidence compared to prospective studies. The last pandemic, Spanish flu, which caused a massive loss of human life all over the world, happened one hundred years ago. None of the current active healthcare workers experienced that pandemic personally. The current pandemic was a unique experience for them. A similarly designed study on 699 adults in the USA showed that sleep data recorded years before the pandemic was closely matched with the sleep data of the pre-pandemic period gathered during the pandemic period [56]. Another study on 7753 adults showed significant changes in lifestyle behaviors during the pandemic compared to the pre-pandemic state [57]. Many lifestyle questions that we asked were about stable habits, and many of the responses were significantly different between the pre-pandemic and pandemic states. Thus, we may assume that subjects, to some extent, were able to recognize changes in their own habits, although we cannot definitely tackle the recall bias, which could be both negative and positive. We used a short questionnaire which limited the amount of information gathered from the healthcare workers. Finally, we believe that implementing new strategies to mitigate the risks associated with declining quality of life among healthcare workers is of the utmost importance, not only in the context of the current pandemic but also as preventative measures for the future. Further studies are needed to illustrate the impact of the current well-being and health index of healthcare professionals on this pandemic’s long-term outcomes.

## 6. Conclusions

In conclusion, the decline in healthcare workers’ well-being and health state during the COVID-19 outbreak could be strongly associated with several lifestyle factors including sleep insufficiency, less physical activity, lower mood state and worse diet. The outcome of this crisis heavily depends on how the pressure of the current pandemic on healthcare workers is addressed, having in mind that they are directly in contact with the afflicted. Comprehensive strategies need to be employed to prevent further deterioration of staff’s quality of life and improve their working conditions.

## Figures and Tables

**Table 1:** Lifestyle questionnaire and the parameters assessed by each question.

Wellness Parameters	Question	Before Pandemic	During Pandemic
Quantity of Sleep	What time did you usually go to bed on weekdays?		
	How long did it take to fall asleep?		
	What time did you usually go to bed on weekends?		
	What time did you usually get out of bed on weekdays?		
	What time did you usually get out of bed on weekends?		
	How many hours did you sleep every night on weekdays?		
	How many hours did you sleep every night on weekends?		
	How many hours did you get a nap on weekdays?		
	How many hours did you get a nap on weekends?		
Sleep Quality	How many days per week did you have difficulties falling asleep?		
	How many days per week did you wake up too early?		
	How many days per week did you use hypnotic medications (sleep aids)?		
	How many days per week did you fall asleep during the day?		
	How many days per week did you feel tired upon waking up in the morning?		
	How many days per week did you snore?		
	How many days per week did you experience mid-sleep awakenings?		
	How many days per week did you experience headaches on awakening?		
	How many days per week did you experience excessive daytime sleepiness?		
	How many days per week did you experience excessive movement during sleep?		
Mood	How many days per week did you experience no energy to get things done?		
	How many days per week did you experience sadness?		
	How many days per week did you experience worry?		
	How many days per week did you experience anger?		
	How many days per week did you experience physical pain?		
Diet	How many days per week did you eat fast food?		
	How many days per week did you eat red meat?		
	How many days per week did you eat fish/omega 3?		
	How many days per week did you eat 4–5 servings of fruits/vegetables?		
	How many days per week did you eat or drink dairy products?		
	How many days per week did you take vitamin D supplements?		
	How many days per week did you take magnesium tablet(s)?		
Physical Activity	How vigorously did you usually engage in pre-planned physical activity? Select one of the following numbers for “Before the start of the current COVID-19 pandemic” and one for “During the current state of the COVID-19 pandemic”: (0) I did not have pre-planned physical activity (1) Very light physical activity (2) Light physical activity (3) Moderately hard physical activity (4) Hard physical activity (5) Very hard physical activity		
	If you selected 1–5, how many minutes in a normal week did you engage in this pre-planned physical activity?		
Social Activity	How many days per week did you participate in a social, cultural or support group that you belong to?		
Smoking	If you smoke, how many cigarettes per day?		
Alcohol	How many drinks of alcohol do you drink in a normal week?		
Wellness and health	How much do you rate your wellness and health out of 20; 20 being the healthiest and 0 being the unhealthiest?		

**Table 2:** Lifestyle characteristics of participants during the pandemic period compared with the pre-pandemic period.

Lifestyle Elements	Before Pandemic		During Pandemic		*p* Value
	Mean	SD	Mean	SD	
Sleep quality score	11.84	9.27	15.24	11.48	0.0001
Mood score	8.35	8.15	11.65	9.39	0.0001
Diet score	13.20	5.07	13.84	4.69	0.001
Physical activity, minutes	23.50	36.20	12.50	24.90	0.0001
Social activity, days per week	2.00	1.80	0.60	0.90	0.0001
Time to go to bed on weekdays	24:20 a.m.	1:22	24:55 a.m.	1:28	0.0001
Time to go to bed on weekends	1:00 a.m.	1:22	1:21 a.m.	1:35	0.0001
Time to fall asleep, minutes	20.50	22.50	24.00	28.30	0.013
Wake-up time on weekdays	7:24 a.m.	1:31	7:48 a.m.	1:43	0.009
Wake-up time on weekends	9:00 a.m.	1:34	9:06 a.m.	1:53	0.342
Night sleep duration on weekdays	7.36	1.68	6.73	1.69	0.274
Night sleep duration on weekends	7.90	1.77	7.69	1.92	0.357
Nap duration on weekdays, minutes	64.8	54.83	69.59	58.53	0.079
Nap duration on weekends, minutes	60.18	57.62	63.44	56.16	0.143
Smoking, pack-year	0.46	2.24	0.30	1.72	0.013
Wellness and health score	15.93	2.99	15.08	3.20	0.0001

**Table 3:** Lifestyle characteristics of participants comparing females and males in the pandemic and pre-pandemic periods.

Lifestyle Elements	Before Pandemic		*p* Value	During Pandemic		*p* Value
	Female	Male		Female	Male	
Sleep quality score, out of 70	13.36	9.71	0.006	17.01	12.65	0.008
Mood score, out of 35	9.88	6.09	0.001	13.02	9.65	0.01
Diet score, out of 49	13.66	12.52	0.1	14.35	13.09	0.06
Physical activity, minutes	19.5	22.3	0.01	8.7	18.2	0.001
Social activity, days per week	1.79	2.21	0.1	0.5	0.7	0.1
Time to go to bed on weekdays	24:19 a.m.	24:21 a.m.	0.9	24:49 a.m.	1:04 a.m.	0.2
Time to go to bed on weekends	24:33 a.m.	1:00 a.m.	0.8	1:17 a.m.	1:29 a.m.	0.4
Time to fall asleep, minutes	22	18	0.2	26.09	20.77	0.2
Wake-up time on weekdays	7:32 a.m.	7:12 a.m.	0.15	7:43 a.m.	7:30 a.m.	0.4
Wake-up time on weekends	9:06 a.m.	8:53 a.m.	0.4	9:07 a.m.	9:03 a.m.	0.8
Night sleep duration on weekdays	7.76	6.69	0.8	6.95	6.49	0.2
Night sleep duration on weekends	7.61	7.59	0.9	7.73	7.64	0.7
Nap duration on weekdays, minutes	67.73	60.61	0.4	78.00	57.22	0.01
Nap duration on weekends, minutes	60.84	59.19	0.8	65.38	60.56	0.5
Smoking, pack/year	0.08	1.01	0.004	0.06	0.65	0.016
Wellness and health score	15.95	15.91	0.9	15.10	15.06	0.9

**Table 4:** Multivariable linear regression models of the self-rated wellness and health index comparing the pre-pandemic and pandemic periods.

	Before Pandemic Model				Covid-19 Pandemic Model			
Lifestyle Elements	β	95% CI for β		*p* Value	β	95% CI for β		*p* Value
		Lower	Upper			Lower	Upper	
Mood score	−0.144	−0.195	−0.093	0.0001	−0.101	−0.147	−0.055	0.0001
Physical activity	0.012	0.001	0.023	0.035	0.023	0.006	0.04	0.01
Diet score	0.078	−0.001	0.157	0.054	0.06	−0.031	0.152	0.20
Wake-up time on weekdays	−0.279	−0.541	−0.018	0.037	−0.183	−0.424	0.059	0.15
Sex	0.731	−0.078	1.54	0.076	Removed by Backward Elimination			
Night sleep duration on weekdays	Removed By Backward Elimination				0.049	0.0001	0.098	0.049
Nap duration on weekdays, minutes					0.009	0.002	0.016	0.01
Age, years					Removed By Backward Elimination			
Sleep quality score								
Time to go to bed on weekdays								
Time to go to bed on weekends								
Time to fall asleep, minutes								
Wake-up time on weekends								
Night sleep duration on weekends								
Nap duration on weekends, minutes								
Snoring								
Social activity								
Smoking, pack/year								
Alcohol								
Comorbid conditions								

## Data Availability

Data are available upon request by contacting the corresponding author.
